# Scleroderma Associated With Organising Pneumonia and Polyarthritis: A Report of a Rare Case

**DOI:** 10.7759/cureus.52886

**Published:** 2024-01-24

**Authors:** Bingu Shiv Kiran Reddy, Babaji Ghewade, Ulhas Jadhav, Pankaj Wagh

**Affiliations:** 1 Pulmonary and Critical Care Medicine, Jawaharlal Nehru Medical College, Datta Meghe Institute of Higher Education and Research, Wardha, IND

**Keywords:** scleroderma, inflammatory polyarthritis, lichen simplex chronicus, frictional dermatitis, organizing pneumonia

## Abstract

Cryptogenic organising pneumonia (COP) is a form of idiopathic diffuse interstitial lung disease (ILD) that develops in response to a variety of unknown irritants. An essential component of the development of organising pneumonia (OP) is damage to type II pneumocytes and the alveolar basement membrane. An autoimmune illness called systemic sclerosis (SSc) has a significant death rate from cardiopulmonary involvement such as pulmonary hypertension and ILD. Arthritis is an autoimmune disorder, in which the patients experience extra-articular symptoms such as ILD during the course of their disease, and COP frequently coexists with these conditions. It is exceedingly uncommon for OP to occur as the initial sign of arthritis, and its clinical characteristics are still unclear. Scleroderma and inflammatory polyarthritis related to COP are presented in this report.

## Introduction

Diffuse interstitial lung illness resulting from damage to the alveolar wall is known as cryptogenic organising pneumonia (COP), the idiopathic type of organising pneumonia (OP). Peripheral lung infiltrates and response to systemic corticosteroid therapy are frequent characteristics. Apart from the cryptogenic variant, secondary OP is linked to autoimmune illnesses like rheumatoid arthritis (RA) and polymyositis, or it can occur following lung radiation exposure, in conjunction with haematological cancers, or as a result of drug exposure. Diffuse alveolar damage, pulmonary haemorrhage, OP, aspiration pneumonia, and lung disease associated with the drugs used in treatment are less common forms of pulmonary involvement reported in systemic sclerosis (SSc). Since there are differences in treatment and a poorer prognosis for a subsequent OP, it is crucial to differentiate from COP [1,2]. There have been only a few reported cases of OP as a manifestation of SSc. Taylor et al. reported only three cases of OP diagnosed by open-lung biopsy in patients with SSc [3]. SSc, also known as scleroderma, is a chronic autoimmune illness marked by skin fibrosis and thickness. It is a connective tissue disease with an unclear aetiology, varying clinical symptoms, a chronic course that frequently progresses, and a tendency to include many organs, including the lungs. About 80% of patients with SSc develop lung fibrosis, and 25% go on to develop progressive interstitial lung disease (ILD), which has a 40% 10-year death rate and is one of the main causes of morbidity and mortality [4].

The involvement of the musculoskeletal system (MSK), including tendinopathy and arthritis, is a typical characteristic of SSc. A combination of joint, tendon, skin, and/or muscle involvement may explain complaints of stiffness and/or discomfort that patients may present with. A variety of joint or muscle inculpations may account for a patient's complaints of pain or stiffness [5]. Here we present a case of OP with active scleroderma associated with frictional dermatitis and lichen simplex.

## Case presentation

A 43-year-old male patient labourer by occupation was admitted inpatient with complaints of breathlessness of Modified Medical Research Council (MMRC) grade II in the last three years, weight loss of around 20 kg in the past two years, and productive cough in the last one month, skin hardening and thickening. He denies a history of smoking and other medical conditions. He has had a known case of inflammatory polyarthritis for the last three years. There was no dust, or smoke exposure in his labourer work, upon general examination, pulse was 98 b/min, respiratory rate was 26/min, blood pressure was 120/80mmhg, SpO2 83% room air, and O2 support 2-3 litre/day for 14-16 hours. Bilateral end-inspiratory crackles of the Velcro variety and coarse crepitations over the left suprascapular area were discovered during the respiratory examination. Other systems were normal. At the time of general examination, the patient also complaint of itching, and lesions over the face for which dermatologist advice was taken as diagnosed as frictional dermatitis and prescribed mometasone furoate cream, tablet hydroxyzine hydrochloride 25mg, the patient also had lesions over the back and was diagnosed as lichen simplex chronicus and prescribed oral terbinafine 250mg for 30 days, bilastine 20mg, sertaconazole nitrate cream, clobetasol propionate cream and mupirocin cream. For the inflammatory polyarthritis and a photosensitive rash over the face, the patient was advised for an anti-cyclic citrullinated peptide (anti-CCP) test by a rheumatologist which was negative, serum calcium was 8.6 mg/dl, and anti-Sjögren's-syndrome-related antigen/anti-Sjögren's syndrome type B was borderline.

**Figure 1:**
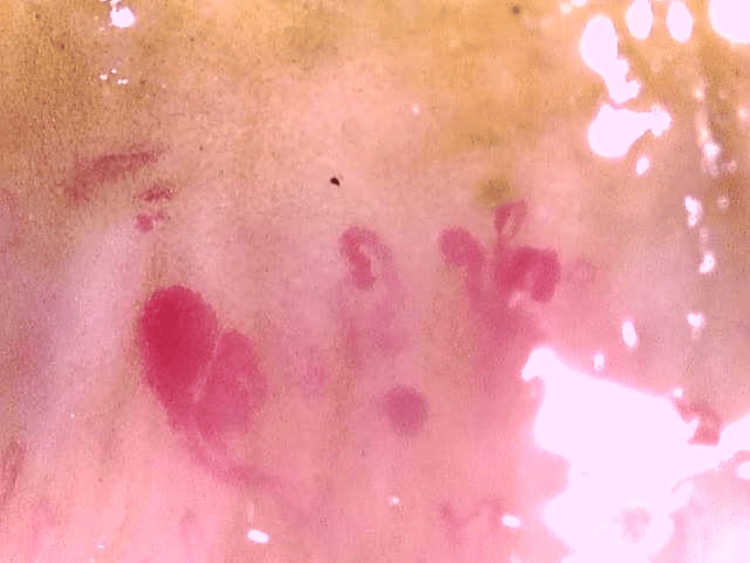
Nailfold Capillaroscopy Showing Dilated Vessels

**Figure 2:**
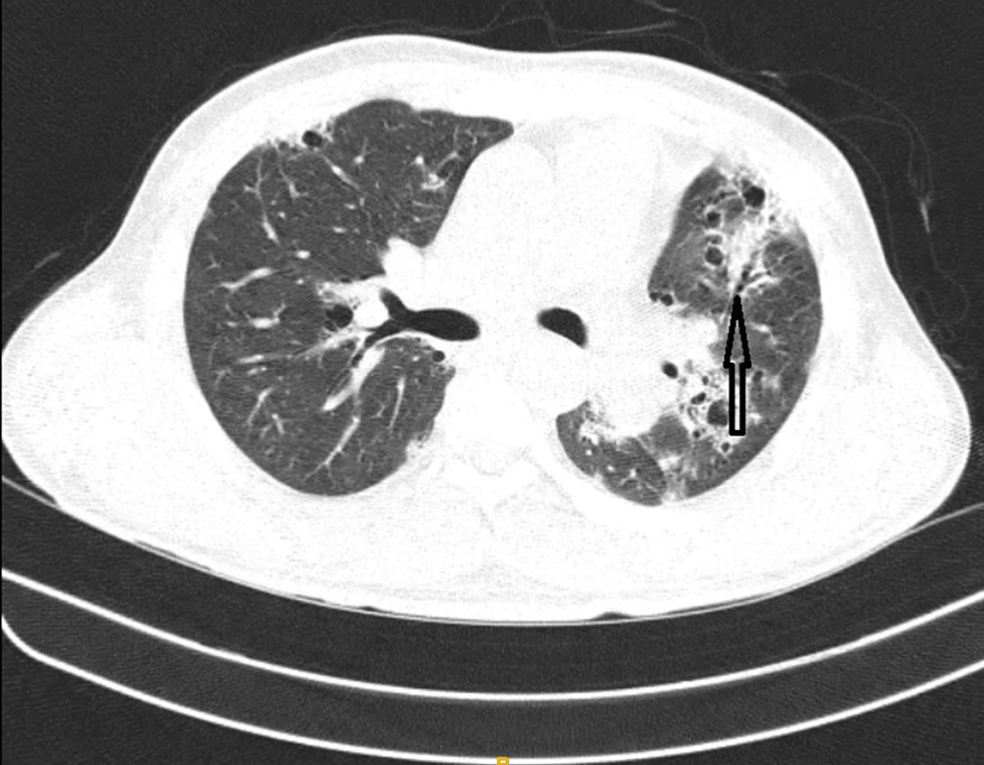
HRCT Showing Patchy Consolidation With Pleural Thickening (Black Arrow) HRCT: High-resolution computed tomography

Nailfold capillaroscopy (Figure 1) was done which showed giant capillaries with micro haemorrhage and areas of capillary loss which was suggestive of an active scleroderma pattern for which the patient has been prescribed tablet Omnacortil 30mg OD for 15 days followed by tablet Omacortil 25mg OD for 15 days, methotrexate 15mg, and tablet folic acid 5mg. Bronchoscopy and bronchoalveolar lavage (BAL) were done to rule out infections, malignancy, and haemorrhage and showed negative for infections (bacterial, viral, fungal, mycobacterial and *Pneumocystis jirovecii* and malignancy. High-resolution computed tomography (HRCT) of the thorax (Figure 2) indicated patchy consolidation with pleural thickening on a lateral segment of the right middle lobe, anterior segment of the left upper lobe, and basal anteromedial segment of left lower lobe; pleural thickening noted in lateral segment of the right middle lobe, basal segments of bilateral lower lobes and superior singular segment of left upper lobe, and multiple centrilobular nodules in left lower lobe. Bronchoscopy and BAL were done to rule out infections, malignancy, and haemorrhage and showed negative for infections (bacterial, viral, fungal, mycobacterial and *P. jiroveciii* and malignancy). The findings were suggestive of OP associated with Ssc. For a conclusive diagnosis, a surgical lung biopsy would be necessary. Following a thorough conversation with the treatment team and himself, the patient made the decision, in light of his radiographic evidence and clinical situation, to proceed with therapy for OP instead of the surgical biopsy. For which the patient was started on 500 mg of azithromycin daily for five days and 60 mg of prednisone, tablet methotrexate 15mg, folic acid 5mg, tablet nintedanib 150mg. The patient showed symptomatically and clinically overall improvement with an O2 saturation of 92% on room air, a week after beginning this treatment plan, and in stable condition, he was discharged from the hospital.

## Discussion

Pathologically speaking, OP is identified by the development of granulation tissue buds in the distal air spaces that progress from exudates of fibrin to fibroblasts which contain collagen [6].

As pneumonia is an inflammatory lung condition, there are several possible causes of OP. Pathologists may describe characteristics of pneumonia that are associated with other conditions like lung cancer, abscess aspiration pneumonia, adult respiratory distress syndrome, pulmonary infarction, and middle lobe syndrome [7].

The pulmonary parenchyma is frequently involved in connective tissue diseases. In this situation, infiltrative lung illness can take many different forms, such as OP, non-specific interstitial pneumonia, or usual interstitial pneumonia. ILD is a type of pulmonary manifestation of SSc, and Ssc-associated OP is extremely rare [8].

Apart from causing pneumonia, connective tissue problems can also result in bronchiolitis obliterans majorly in RA. Despite the fact that OP can have a variety of causes or manifest in conjunction with systemic illnesses, it is usually cryptogenic and isolated [9].

First-line diagnostic procedures for COP include chest radiographs followed by HRCT of the chest, which frequently reveal diffuse patchy consolidations involving bilateral lower zones as well as bilateral patchy peripherally situated consolidations. The bronchoscopy and BAL are usually the next steps in the diagnostic workup. A surgical lung biopsy is necessary to provide a definitive diagnosis. Preferably, wedge biopsies should come from at least two lobes where there is radiographic evidence of COP involvement. However, the quality of the patient's clinical picture must be considered when comparing the risks of these surgical techniques. If the clinical and radiological data is compatible with COP, treatment can begin without performing a biopsy of the lung after consulting with the patient [10].

There are times when OP results in spontaneous improvement [11,12]. Other individuals have shown only little improvement with extended erythromycin treatment [13]. Nonetheless, corticosteroids are currently the accepted standard of care, while it is less clear what dosage and duration are best for full recovery. Corticosteroids cause a noticeably less intense reaction in patients with idiopathic chronic eosinophilic pneumonia [14]. Clinical signs typically recover in 48 hours, but radiographic lung infiltrates typically take several weeks to completely resolve (usually without significant sequelae). Most patients improve noticeably after just one week of therapy [15-17].

## Conclusions

In this case study, a 43-year-old patient with distinct radiographic characteristics of OP, an unusual lung disease, is highlighted and successfully treated. This case study further emphasizes the scant evidence linking OP to autoimmune diseases which was an accidental finding and skin lesions, specifically lichen simplex chronicus and frictional dermatitis. This is a rare association of diseases; scleroderma along with associated OP and polyarthritis should be diagnosed properly and treated accordingly.
